# A Novel 3D Approach with a CNN and Swin Transformer for Decoding EEG-Based Motor Imagery Classification

**DOI:** 10.3390/s25092922

**Published:** 2025-05-05

**Authors:** Xin Deng, Huaxiang Huo, Lijiao Ai, Daijiang Xu, Chenhui Li

**Affiliations:** 1 Chongqing Key Laboratory of Germplasm Innovation and Utilization of Native Plants, Chongqing 401329, China; 2 The Key Laboratory of Data Engineering and Visual Computing, Chongqing University of Posts and Telecommunications, Chongqing 400065, China

**Keywords:** brain–computer interface (BCI), electroencephalogram (EEG), motor imagery (MI), 3D convolutional neural network (3D CNN), 3D Swin Transformer

## Abstract

Motor imagery (MI) is a crucial research field within the brain–computer interface (BCI) domain. It enables patients with muscle or neural damage to control external devices and achieve movement functions by simply imagining bodily motions. Despite the significant clinical and application value of MI-BCI technology, accurately decoding high-dimensional and low signal-to-noise ratio (SNR) electroencephalography (EEG) signals remains challenging. Moreover, traditional deep learning approaches exhibit limitations in processing EEG signals, particularly in capturing the intrinsic correlations between electrode channels and long-distance temporal dependencies. To address these challenges, this research introduces a novel end-to-end decoding network that integrates convolutional neural networks (CNNs) and a Swin Transformer, aiming at enhancing the classification accuracy of the MI paradigm in EEG signals. This approach transforms EEG signals into a three-dimensional data structure, utilizing one-dimensional convolutions along the temporal dimension and two-dimensional convolutions across the EEG electrode distribution for initial spatio-temporal feature extraction, followed by deep feature exploration using a 3D Swin Transformer module. Experimental results show that on the BCI Competition IV-2a dataset, the proposed method achieves 83.99% classification accuracy, which is significantly better than the existing deep learning methods. This finding underscores the efficacy of combining a CNN and Swin Transformer in a 3D data space for processing high-dimensional, low-SNR EEG signals, offering a new perspective for the future development of MI-BCI. Future research could further explore the applicability of this method across various BCI tasks and its potential clinical implementations.

## 1. Introduction

Brain–computer interface (BCI) technology, which serves as a direct communication pathway between the brain and external devices, has become a key field in scientific research and clinical applications [1]. This technology not only offers a new communication and control pathway for patients with limited motor functions [2] but also demonstrates vast potential in fields such as motor rehabilitation, emotion recognition, and human–computer interactions [3,4,5]. Among non-invasive BCI technologies, electroencephalogram (EEG) is favored for its non-invasiveness, low cost, and high portability. EEG captures brain activity through electrodes placed on the scalp, and its signals can be decoded to control external devices such as computers, wheelchairs, or robots [6,7]. However, the variability of EEG signals and their susceptibility to external interference pose significant challenges in extracting useful information from noisy backgrounds.

Traditional pattern recognition techniques, such as the common spatial pattern (CSP) method and its variants [8,9,10], excel in enhancing spatial features related to motor imagery (MI) tasks; however, they often rely on task-specific prior knowledge, limiting their generalization capabilities. Time–frequency analysis methods, such as continuous wavelet transform (CWT) [11] and empirical wavelet transform (EWT) [12], can extract rich time–frequency features, but the applicability of these traditional methods is limited in different BCI paradigms.

In recent years, deep learning technologies, especially convolutional neural networks (CNNs) [13] and recurrent neural networks (RNNs) [14], have gained attention for their potential in feature learning and time-series data classification. CNNs have demonstrated powerful representational capabilities in computer vision tasks, while RNNs and long short-term memory (LSTM) networks [15] have been designed to capture long-term dependencies in time-series data. Some studies combine CNNs and LSTMs to leverage the strengths of both model types [16], or use CNNs with multiple convolutional kernel scales [17] for better classification results. However, CNNs are limited in capturing global temporal dependencies due to their local receptive fields, and RNNs inherently struggle with maintaining long-term dependencies and parallel training.

The Transformer model has attracted attention for its excellent performance in natural language processing and image recognition. Its self-attention mechanism effectively captures long-distance dependencies [18], providing a new solution for EEG signal decoding [19]. However, the application of Transformer models in EEG signal processing is still in its infancy, especially in effectively integrating global and local feature learning. There is a lack of comprehensive analysis and visualization studies on the role of Transformers in EEG signal decoding.

To address these problems, we propose an innovative EEG data representation method, which transforms the raw EEG signals into a three-dimensional structure containing time dimensions and a two-dimensional EEG electrode distribution, with delta, theta, alpha, beta, and gamma frequency ranges as different channels. Based on this, we design a deep learning framework that employs one-dimensional CNNs to process time dimensions and two-dimensional CNNs to handle the electrode plane, to capture local spatio-temporal features. Subsequently, we introduce a window-based self-attention mechanism—the 3D Swin Transformer [20,21]—to extract global features in EEG signals.

The contributions of this paper are as follows:

- We propose a novel and effective Convolution Swin Transformer (ConSwinFormer) network for rapid feature extraction of EEG signals for MI task classifications.
- Extensive experiments are conducted to research the impact of the Swin Transformer module and attention parameters.
- Data augmentation techniques, including segmentation and reconstruction (S&R) and random noise addition, effectively improve the model’s generalization ability on small datasets and reduce the risk of overfitting.
- Through spatio-temporal feature map visualization analysis, we enhance the interpretability of the model, providing new insights into brain region activities and collaborative work during the MI process.

The subsequent sections of this paper first detail the experimental design, model structure, training procedure, and result analysis. Then, we discuss the application of the model in different types of MI tasks and the visual interpretation of the decision-making process. Finally, future research directions and potential applications are proposed. Through this research, we expect to provide new insights into the application of deep learning in the BCI domain.

## 2. Related Work

### 2.1. CNN, RNN, LSTM

In the research on MI within BCI, deep learning methods play an important role, such as CNNs, RNNs, and LSTMs. CNNs are renowned for their capability to extract spatial features from EEG signals, making them highly effective for tasks requiring the recognition of complex spatial patterns inherent in MI data. For instance, Li et al. [22] proposed a CNN-LSTM feature fusion network, demonstrating that CNNs can effectively extract spatial features, and LSTMs are helpful in capturing long-term dependencies in time-series data. Additionally, Liu et al. [23] proposed a multi-branch one-dimensional convolutional neural network (CMO-CNN), which uses multiple convolutional kernels to extract features from EEG at different time scales, providing a compact and effective solution. On the other hand, RNNs and LSTMs excel in capturing temporal dependencies, which is crucial for understanding the sequential nature of EEG signals in MI tasks. Supakar et al. [24] proposed a model that demonstrated the efficacy of RNN-LSTMs in processing EEG signals, especially in identifying complex conditions such as schizophrenia. By enabling a more detailed and comprehensive analysis of brain signals, these methods significantly enhance the accuracy and robustness of MI-BCI systems. In a review research on deep learning techniques for classifying EEG signals, Altaheri et al. [25] also noted that deep learning technologies, particularly CNNs and LSTMs, have been extensively applied in MI-BCI systems, demonstrating their powerful capability in processing such signals.

### 2.2. Three-Dimensional CNN

The emergence of 3D convolutional neural networks (3D CNNs) marks a significant development in the field of convolutional neural networks. Three-dimensional CNNs extend the capabilities of traditional CNNs by adding additional dimensions. This multidimensional approach is particularly beneficial for MI classification tasks. As both spatial and temporal correlation information of brain activity are crucial, 3D CNNs allow us to process spatial and temporal information in EEG signals simultaneously to obtain their correlation information. Zhao et al. [26] proposed a multi-branch 3D CNN model specifically for the classification of EEG signals, effectively enhancing task performance. Similarly, the BrainGridNet model proposed by Wang et al. [17], a two-branch deep convolutional network, demonstrated the effectiveness of 3D CNNs in decoding multi-class MI tasks based on EEG signals. Park et al. [27] also introduced a model based on 3D CNNs, which aims to improve the accuracy and interpretability of MI classification through a deep analysis of spatio-temporal features. The multi-branch architecture of these networks further enhances their capability to process different patterns in EEG data, thus improving the performance of MI classification tasks and offering strong robustness and adaptability across different subjects and sessions. Li and Ruan [28] also confirmed the effectiveness of novel decoding methods using 4D data representation and 3D CNNs in MI tasks, demonstrating the potential of 3D CNNs in processing complex EEG signals.

### 2.3. Transformer

With its unique advantage of self-attention mechanisms, the Transformer model is gradually replacing traditional deep learning models as a powerful tool in the field of EEG signal processing. This model structure can effectively capture the long-term dependencies and the complex patterns in EEG signals, being particularly effective for decoding time-series MI data. The self-attention mechanism, by assigning different attention weights to each part of the input data, allows the model to focus on signal features most relevant to the current task. For example, Xie et al. [29] proposed the Transformer model based on learning the spatio-temporal features of EEG decoding, demonstrating its potential in EEG signal processing. Luo et al. [30] proposed the SMT model, which improved the accuracy and generalization ability of cross-subject EEG signal classification by introducing multiple self-attention mechanisms and mirror network structures. Similarly, Deny et al. [31] proposed a Hierarchical Transformer model specialized for MI-BCI, confirming that the Transformer is useful in understanding the effectiveness of complex brain electrical signals. Moreover, Ma et al. [32] proposed a CNN-Transformer model, also showing its strong performance. The ability to capture the long-term dependencies enables Transformers to not only identify significant local features but also to perceive the correlations across different time and spatial regions, revealing the subtle changes in the brain during the execution of specific tasks.

### 2.4. Swin Transformer

The Swin Transformer is an innovative architecture that builds on the traditional Transformer. Its main advantage is that it can learn local details and global information at the same time, so it shows high efficiency in decoding EEG signals. The Swin Transformer improves the self-attention mechanism by introducing a hierarchical structure and shifted window strategy, allowing the model to capture features at various resolutions. This enables it to precisely identify complex patterns in EEG signals. For instance, Xu et al. [33] proposed Swin-TCNet, a time–frequency channel cascade network based on the Swin Transformer, demonstrating the superiority of the Swin Transformer in processing MI intracranial electroencephalography signals. Similarly, Wang et al. [34] proposed a new algorithm combining EEG channel attention and the Swin Transformer to enhance the performance of MI paradigm classification. Additionally, Li et al. [35] proposed the MST-Net, a multi-scale Swin Transformer network for EEG-based cognitive load assessments, which also demonstrated the effectiveness of the Swin Transformer in complex EEG signal decoding. This structure not only enhances the model’s sensitivity to minor signal variations but also improves its recognition of broad patterns of brain activity, significantly boosting the classification accuracy and computational efficiency of MI-BCI systems.

## 3. Methods

**Figure 1:**
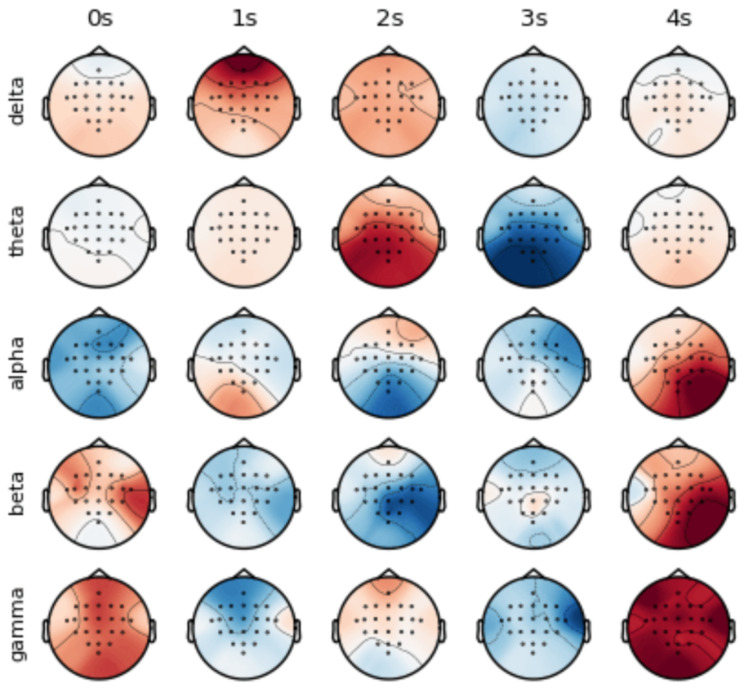
Visualization of spatio-temporal frequency features in raw EEG signals from Subject 01.

Neural activities show the characteristics of asynchronous excitation in different brain regions [36]. As shown in Figure 1, different times, frequency bands and channels show unique activation patterns. In order to utilize the information of the time–frequency domain and channel, we propose the ConSwinFomer, an end-to-end spatio-temporal frequency feature extraction network that fuses a CNN and Swin Transformer. The ConSwinFomer contains four main parts: preprocessing module, CNN module, Swin Transformer [20,21] module, and classifier module.

### 3.1. Overview

**Figure 2:**
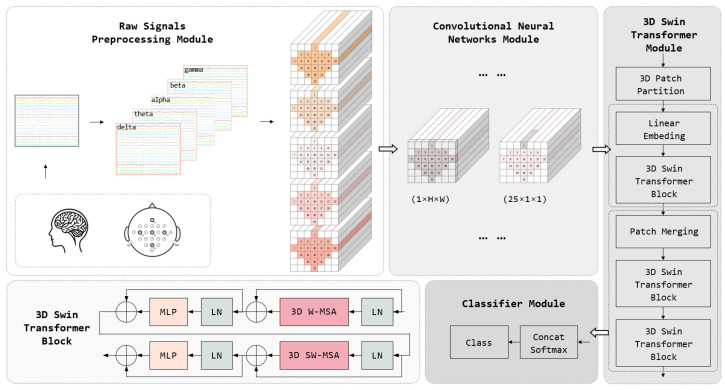
The overall architecture of ConSwinFormer, with details of the 3D Swin Transformer block in the lower left corner of the Transformer module.

Our model takes raw EEG signals as the input, processes the channel and time-point data dimensions, and outputs the probability distribution for different MI task categories. The model aims to utilize the local feature extraction capability of CNNs and the global correlation feature extraction ability of the Swin Transformer to achieve effective feature extraction of three-dimensional EEG data in spatio-temporal frequency dimensions. The workflow of the ConSwinFormer, as shown in Figure 2, begins with the preprocessing stage, where EEG data are divided into multiple frequency bands. These data are then structured into a three-dimensional construct according to the spatial location of electrodes on the scalp and the time dimension, aiming to capture the spatio-temporal frequency characteristics of EEG signals. Following this, the CNN module extracts local features, identifying the local temporal and spatial patterns and laying the foundation for subsequent global feature extraction. The Swin Transformer module enhances the model’s global feature representation by learning feature interactions within different windows through skip connections. Finally, the classifier module outputs probabilities for MI tasks based on the extracted features, converting complex feature information into specific classification results. Overall, by integrating the characteristics of CNNs and the Swin Transformer, our model significantly improves the classification accuracy of MI tasks in processing 3D EEG data.

### 3.2. Preprocessing Module

In this research, the preprocessing stage plays a crucial role in ensuring that the acquired data are of high quality and are suitable for real-time processing. By using the label information collected during data acquisition, we segment the raw EEG data into several trials, each structured in an N×D format, with the *N* representing the number of electrode channels and *D* denoting the number of time points, equivalent to the sampling time multiplied by the sampling frequency. During preprocessing, we implement a series of simplification and efficient steps aimed at ensuring data quality while considering computational efficiency and latency for real-time tasks. These steps include filtering, normalizing, and rearranging the data. Some complex calibration and artifact removal methods that may cause processing delays are intentionally omitted. It is expected to achieve fast processing speeds and low response delays in real-time tasks.

The EEG data undergo band-pass filtering to obtain brain signals in five different frequency ranges. These frequencies include δ (0.5–4 Hz), θ (4–8 Hz), α (9–12 Hz), β (13–30 Hz), and γ (31–50 Hz), each corresponding to different states of brain activity. The primary rhythms related to MI tasks are mu (μ) and beta (β) [37,38], with the μ rhythm overlapping with the α band. By employing band-pass filters, we effectively remove the high-frequency and low-frequency noise unrelated to the research. After filtering, the data format is transformed into C×N×D, where *C* represents the number of frequency bands. Specifically, we use *C*, which denoted finite impulse response filters (FIRs), aligning with many related studies [28,29,32], aiming to retain only the specific frequency ranges of interest. Subsequently, these *C* frequency bands are the input into the model as different data channels for further analysis and classification.

Z-score normalization was applied to the band-pass filtered EEG data to reduce volatility and nonstationarity in the data. This process was completed using the following equation:

(1) $$X=\frac{x-\mu }{\sqrt{\sigma^{2}}},$$

In Equation (1), *x* represents the original band-pass filtered data, while *X* is the output data after normalization. μ and $\sigma^{2}$ represent the mean and variance of these data, respectively. Through such processing, each data point is adjusted to ensure better consistency and comparability across different trials and channels.

**Figure 3:**
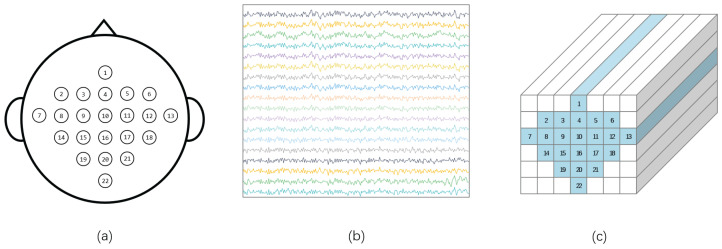
(**a**). Schematic representation of electrode spatial locations for EEG acquisition (**b**). Original EEG data arrangement (N×D) (**c**). Rearranged EEG data distribution (D×H×W).

The two-dimensional EEG data are rearranged according to the actual locations of electrodes on the scalp, transforming them into a three-dimensional structure. This arrangement helps more effectively extract spatio-temporal features between adjacent electrodes. Based on the research by Zhao et al. [26], this 3D representation method outperforms the traditional two-dimensional representation in decoding performance, and the research by Zhang et al. [39] also shows that 3D EEG has better decoding performance than 2D EEG. Specifically, we rearrange the two-dimensional EEG data according to the spatial distribution of electrodes on the scalp. As illustrated in Figure 3c, this rearrangement transforms the signal into a D×H×W data format, where *H* and *W* represent height and width, respectively, covering all *N* channels. Figure 3a shows the layout of electrodes on an EEG cap, while Figure 3b displays the arrangement of N×D EEG data for a trial. After filtering and rearrangement, the data dimensions become C×D×H×W. This data structure provides a comprehensive perspective for subsequent feature extraction and classification efforts, enabling the model to efficiently utilize the spatio-temporal frequency features in EEG signals.

### 3.3. CNN Module

In our proposed model, the CNN part plays a crucial role and is mainly used to extract local features in EEG signals. Research over recent years has confirmed the efficiency of CNNs in capturing local features [22,23,25,26,27,28]. The first step in the model is to use a CNN for carrying out the preliminary local feature extraction from EEG signals, aiming at capturing local spatio-temporal patterns within EEG data, preparing for subsequent global feature extraction and classification tasks. Inspired by existing research [40], our model adopts an innovative approach to decompose spatio-temporal 3D convolutions into 2D spatial convolutions and 1D temporal convolutions. The two-dimensional convolution uses kernels of size (1,H,W) with padding set to (0,⌊H/2⌋,⌊W/2⌋), allowing the convolution kernel to act as a spatial filter, capturing the relationships between electrode channels on a single time sample. The spatial convolution kernel is directly applied to the entire electrode plane. One-dimensional temporal convolution uses kernels of size (25,1,1) with padding set to (12,0,0), serving as a temporal filter that aims to capture local features along the time dimension for individual channels. The number 25 in the convolution kernel represents a 0.1-second sampling window, which aids in capturing minor changes over time. In order to improve the stability and generalization ability of the model, we add batch normalization after each convolutional layer. This not only helps prevent the vanishing gradient problem but also speeds up the training process and improves the adaptability of the model to different input data. Overall, the CNN module effectively extracts the key local features in EEG signals by combining convolution in spatial and temporal dimensions, which lays a solid foundation for the subsequent global feature extraction and classification tasks of the model.

### 3.4. Three-Dimensional Swin Transformer Module

After the preliminary extraction of spatio-temporal local features in EEG signals through the CNN module, to explore the global connections between these local features more deeply, we adopt the hierarchical Swin Transformer module [20,21]. The architectural design of the Swin Transformer module enables us to perform effective feature extraction at various scales and enhances feature extraction capabilities through stacking multiple 3D Swin Transformer modules at specific scales.

The 3D Swin Transformer module comprises two primary blocks, each slightly differing in their application of the Multi-Head Self-Attention (MSA) mechanism. The structures of these blocks include Layer Normalization (LayerNorm) [41], 3D Window Multi-Head Self-Attention (3D W-MSA) or 3D Sliding Window Multi-Head Self-Attention (3D SW-MSA), LayerNorm again, and a Multi-Layer Perceptron (MLP). Before performing MSA calculations, the input sequence is first processed with LayerNorm, followed by the output of MSA being residually connected to the output of the previous layer and then processed through LayerNorm again before being input into the MLP.

**Figure 4:**
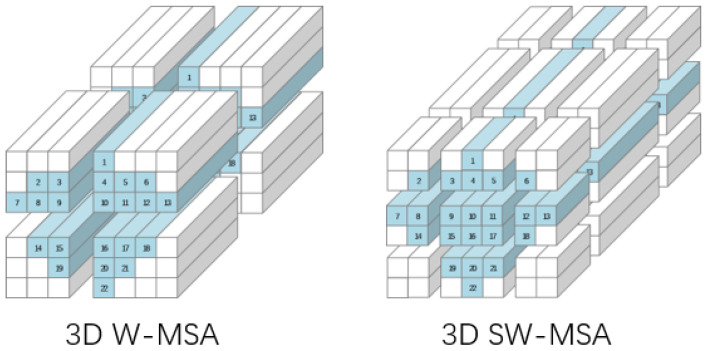
Window attention mechanism and sliding window attention mechanism.

In the first block of the 3D Swin Transformer module, 3D W-MSA with a fixed window position is used to construct attention weights for local positions. The second block adopts the 3D SW-MSA mechanism, as shown in Figure 4, which enables global dependency construction across windows by moving the window to a position between several windows of the previous block. Such a design allows the 3D Swin Transformer module to capture not only the interactions between local features but also to understand broader global dependencies.

By stacking 3D Swin Transformer block at different scales, the model can more comprehensively convey dependency information between windows, thereby constructing a richer global attention parameter set. This hierarchical 3D Swin Transformer module design is important for decoding global spatio-temporal frequency dependencies in EEG signals, effectively enhancing the accuracy of MI task classification.

#### 3.4.1. Multi-Head Self-Attention Mechanism

The ConSwinFormer model uses the 3D Swin Transformer module designed based on the Transformer architecture. One of the core features of Transformer is the MSA mechanism, which processes input information at different locations in multiple subspaces by using multiple attention heads. Each attention head produces a unique attention distribution that allows the model to efficiently integrate and process a large amount of subspace information from different locations. In our model, the MSA is used by dividing the data into windows with intersections through 3D W-MSA and 3D SW-MSA, aiming to extract global dependencies in 3D EEG signals.

The core working principle of the MSA mechanism is to map input features to multiple subspaces and perform the dot product operation on these subspaces to calculate the corresponding attention vector [18]. These attention vectors, computed in each subspace, are then combined together and mapped back to the original input space to form the final output vector. This mechanism greatly enhances the ability of the model to handle complex inputs, especially when analyzing 3D EEG signals with rich spatio-temporal properties.

The MSA mechanism is described by the following equations:

(2) $$\mathrm{MSA}\left(Q,K,V\right)=\mathrm{concat}\left({\mathrm{head}}_{1},\ldots ,{\mathrm{head}}_{N_{h}}\right)W^{o},$$

(3) $${\mathrm{head}}_{i}=\mathrm{Attention}\left(QW_{i}^{Q},KW_{i}^{K},VW_{i}^{V}\right),$$

(4) $$\mathrm{Attention}\left(Q_{i},K_{i},V_{i}\right)=\mathrm{Softmax}\left(\frac{Q_{i}K_{i}^{T}}{\sqrt{d_{k}}}\right)V_{i},$$

where $head_{i}$ represents the *i*-th attention head, and $N_{h}$ is the total number of attention heads. $W^{o}\in R^{hd_{v}\times d_{\mathrm{model}}}$ is a linear mapping matrix used to merge the outputs from all attention heads. $QW_{i}^{Q}$, $KW_{i}^{K}$, and $VW_{i}^{V}$ denote the linear mapping of the query (Q), key (K), and value (V) for the *i*-th attention head, respectively. $W_{i}^{Q}\in R^{d_{\mathrm{model}}\times d_{q}}$, $W_{i}^{K}\in R^{d_{\mathrm{model}}\times d_{k}}$, and $W_{i}^{V}\in R^{d_{\mathrm{model}}\times d_{v}}$ are the weight matrices for the query, key, and value, respectively. $d_{q}$, $d_{k}$, and $d_{v}$ refer to the dimensions of the query, key, and value, respectively.

Through this MSA mechanism, the ConSwinFormer model effectively captures and integrates features from different times, frequency bands, and spatial positions of EEG signals, significantly enhancing the decoding capability for complex EEG signals.

#### 3.4.2. Three-Dimensional W-MSA

The 3D Window Multi-Head Self-Attention (3D W-MSA) serves as a cornerstone in the ConSwinFormer model, primarily for extracting local dependencies within three-dimensional input vectors. It operates by calculating attention scores within fixed-size windows to capture intricate spatio-temporal features in each local window [42]. The input for 3D W-MSA consists of 3D spatio-temporal EEG data under specific frequencies, with the input feature vector dimension being (C,D,H,W), where *C* represents frequency channels, *D* is the time dimension, and *H* and *W* denote the height and width of the electrode plane, respectively. These feature vectors are partitioned into non-overlapping windows of size (d,h,w), forming (C,D/d,H/h,W/w) independent local windows.

As illustrated in Figure 2, the input feature vector of dimensions (C,D,H,W) is divided into (C,T/d,H/h,W/w) non-overlapping windows, within each of which the 3D W-MSA performs self-attention operations. This implies that the model assesses the interactions and relationships among different parts within a window, thereby capturing local spatio-temporal characteristics. Attention scores for each window are calculated independently, aiding the model in understanding the complex associations between local features.

The window size in 3D W-MSA is statically configurable, allowing for adjustments based on varying experimental setups and data characteristics to optimize model performance. The computation process [21] is represented by the following equations:

(5) $${\hat{z}}^{l}=3D\_\mathrm{W-MSA}\left(\mathrm{LN}\left(z^{l-1}\right)\right)+z^{l-1},$$

(6) $$z^{l}=\mathrm{FFN}\left(\mathrm{LN}\left({\hat{z}}^{l}\right)\right)+{\hat{z}}^{l},$$

where ${\hat{z}}^{l}$ and $z^{l}$ represent the output features of the 3D W-MSA block and the feed-forward network (FFN) at the *l*-th layer, respectively, with LN denoting layer normalization. Three-dimensional W-MSA signifies Multi-Head Self-Attention based on three-dimensional windows using a fixed window partition. This design enables the model to efficiently capture and comprehend local spatio-temporal features in 3D EEG data, thus enhancing decoding capabilities for complex EEG signals.

#### 3.4.3. Three-Dimensional SW-MSA

The 3D Sliding Window Multi-Head Self-Attention (3D SW-MSA) is an important component of the ConSwinFormer model, aimed at strengthening the connections between local windows. Unlike 3D W-MSA, which limits itself to computing self-attention within individual local windows, 3D SW-MSA facilitates information exchange between windows through a sliding window mechanism, capturing a broader range of global dependencies.

In the 3D Swin Transformer module, 3D SW-MSA follows the 3D W-MSA block, enhancing inter-window connections by shifting local windows. This mechanism employs half the length of the original patch as the step size for window movement, enabling the model to apprehend dependencies across multiple windows. The self-attention computation based on the sliding window mechanism is conducted following the window shift operation, allowing 3D SW-MSA to understand not only the information within individual local windows but also the inter-relations between windows.

Like 3D W-MSA, the window size in 3D SW-MSA is also statically configurable, offering researchers the flexibility to adjust the window sizes based on the specific datasets and experimental requirements. The specific computation process [21] is given by the following equations:

(7) $${\hat{z}}^{l+1}=3D\_\mathrm{SW-MSA}\left(\mathrm{LN}\left(z^{l}\right)\right)+z^{l},$$

(8) $$z^{l+1}=\mathrm{FFN}\left(\mathrm{LN}\left({\hat{z}}^{l+1}\right)\right)+{\hat{z}}^{l+1},$$

where ${\hat{z}}^{l}$ and $z^{l}$, respectively, denote the output features of the 3D SW-MSA block and the feed-forward network (FFN) block at the *l*-th layer, with LN representing layer normalization. Three-dimensional SW-MSA, as the Multi-Head Self-Attention based on 3D window shifting, utilizes sliding window partition. Through this design, 3D SW-MSA in the ConSwinFormer model bolsters the capture of global dependencies in complex EEG signals, supporting deeper feature extraction and precise classification.

#### 3.4.4. Hierarchical Structure

In the ConSwinFomer model, the 3D Swin Transformer module plays a crucial role in processing and analyzing the complex spatio-temporal features in 3D EEG signals. Our designed model comprises two layers of 3D Swin Transformer blocks, with the first layer including one 3D Swin Transformer block, and the second layer being composed of two Swin Transformer blocks. This hierarchical structure enables the model to capture more detailed features across different scales.

After initial feature extraction in the CNN stage, the 3D Swin Transformer module employs the 3D Patch Partition technique to divide 3D EEG data into multiple small patches of size d×h×w, each considered as an independent token with a 96-dimensional feature representation. This division facilitates more precise processing of local information in EEG signals.

Subsequently, these divided patches undergo linear embedding to be transformed into a format efficiently processable by the model. For input features of size (C,D,H,W), the 3D Swin Transformer module outputs features of different sizes after two stages, thereby achieving feature extraction across various scales.

Each 3D Swin Transformer module contains a 3D W-MSA block that calculates within fixed-size windows, focusing on capturing local attention weights. Additionally, the 3D SW-MSA enhances inter-window communication through window shifting operations, thereby capturing global dependencies. Under this mechanism, windows move half the distance to the right and downward, followed by the attention weight calculation in the new window positions.

#### 3.4.5. Three-Dimensional Relative Position Bias

The ConSwinFomer model introduces the concept of a 3D relative position bias, based on findings from previous research that, considering the relative position bias for each head in self-attention calculations, can enhance model performance [43]. In this model, a 3D relative position bias is introduced for each attention head, represented as a three-dimensional tensor $B\in R^{P^{2}\times M^{2}\times M^{2}}$, where *P* and *M*, respectively, stand for the dimensions of the temporal and spatial domains (height or width). Specifically, the self-attention mechanism considers this relative position bias, formulated as

(9) $$\mathrm{Attention}\left(Q,K,V\right)=\mathrm{SoftMax}\left(QK^{T}/\sqrt{d}+B\right)V.$$

where $Q,K,V\in R^{PM^{2}\times d}$ represent the query, key, and value matrices, respectively, and *d* is the dimensionality of these features. $PM^{2}$ is the number of tokens in a 3D window. Since the relative position along each axis lies within the range [−P+1,P−1] (temporal) or [−M+1,M−1] (height or width), a smaller bias matrix $\hat{B}\in R^{\left(2P-1)\times (2M-1)\times (2M-1\right)}$ is used in the model to simplify computations. In this setup, the values of $\hat{B}$ are directly extracted from the original position bias matrix *B*.

## 4. Experiments and Results

### 4.1. Dataset

To evaluate the performance of the proposed ConSwinFormer model, we selected the BCI Competition IV 2a(BCICIV-2a) dataset provided by Graz University of Technology [44] as the benchmark. This dataset is one of the most commonly used and widely recognized benchmarks in the field of MI classification, making it suitable for our assessment purposes.

The BCICIV-2a comprises EEG data from 9 subjects. Each subject participated in four different MI tasks, including imagining movements of the left hand, right hand, both feet, and tongue. These diverse tasks place high demands on the classification ability of the model, making this dataset an ideal evaluation tool.

Data were collected using 22 Ag/AgCl electrodes and 3 electrooculogram (EOG) electrodes, carefully positioned to maximize the signal capture range and quality. All data were recorded at a sampling rate of 250 Hz, processed through a band-pass filter of 0.5 to 100 Hz. This high-quality data collection and preprocessing ensure the consistency and reliability of the input data.

In addition, another feature of this dataset is that it contains two sets of data collected by each subject at different times to evaluate the cross-session ability of the model, which makes the experimental design more challenging and realistic. Each session contained 288 trials, equally divided among the four tasks, i.e., 72 trials per task. This type of data distribution provides a balanced dataset for the task.

In the data preprocessing, we specifically focused on the 2∼6.5 s interval of each trial and further applied a band-pass filter to filter the EEG data to the frequency range of 0.2∼40. Such data processing steps aim to optimize the input data, making them more suitable for deep learning model processing. Data from each subject were divided into ten parts, with nine parts used for training the model and the remaining part used for model evaluation. This process was repeated 10 times, which is called 10-fold cross-validation.

Overall, the application of BCICIV-2a provides us with a comprehensive, challenging, and representative platform to evaluate and demonstrate the classification performance of our proposed ConSwinFormer model in MI tasks.

### 4.2. Data Augmentation

During the collection of EEG data, one challenge we faced was the small size of the dataset, which was due to the complexity of the acquisition process itself and the time cost required. Training deep learning models on such small datasets commonly results in overfitting, where the model performs excellently on the training set but poorly on the validation set. To overcome overfitting, we adopt time-domain-based data augmentation techniques. One approach utilized to generate new data samples is the Segment and Reassemble (S&R) technique [45]. The operation involves dividing EEG data of the same category into multiple segments, each containing consecutive time points, and then randomly reassembling these segments while maintaining the original time sequence, creating new data samples. This strategy not only utilizes the time sequence information in the original data but also generates diversity through reassembly, effectively enlarging the dataset. Moreover, we introduce random noise into the reconstructed samples, further enhancing data variability and improving the model’s adaptability and robustness to unknown data. During the training phase, we employ a dynamic data augmentation strategy. This means that, for each training iteration, we generate augmented data samples equal to the batch size, ensuring the model encounters new transformed data in every training cycle. This approach not only enriches the diversity of the training data but also strengthens the model’s generalization ability against varied test data. This dynamic and diversified training method effectively reduces the risk of model overfitting, enhancing the model’s accuracy and stability when processing real-time EEG data.

### 4.3. Experimental Details

Our research method was implemented in a computing environment equipped with an RTX 3090 GPU (Nvidia, Santa Clara, CA, USA), using Python 3.11 and the PyTorch 2.1 library [46] for programming and model construction. During the data preprocessing stage, we first removed the EOG signal channels from the dataset and then applied a band-pass filter to process the EEG signals. Beyond basic filtering steps, no additional artifact removal measures were taken. It is worth mentioning that the direct removal of EOG data was due to the following reasons: Most of the existing methods using EOG are performed to eliminate electroencephalogram artifacts in other channels through ICA. However, MI is a real-time task, and it is difficult to apply the electroencephalogram proposed by ICA in real-time tasks. If the EOG channel data are directly connected to the model for processing, this will result in additional complexity, which will make it more difficult for the model to learn useful features.

Model training was conducted based on data specific to each participant, a common practice in the BCI field. To ensure the reliability and generalizability of the evaluation results, we adopted the ten-fold cross-validation method. Specifically, for each participant, the training set included 520 trials, and the test set included 56 trials.

During model training, we utilized the Adam optimizer [47] to optimize model parameters, aiming to minimize the cross-entropy loss function. Our carefully selected hyperparameters aimed to achieve optimal performance, with the batch size being set to 18, learning rate fixed at 1 $\times \;10^{-4}$, and the number of iterations set to 200. To ensure the model could learn effectively and reach convergence, the selection of these parameters was based on initial testing and validation experiments.

### 4.4. Experimental Results

**Figure 5:**
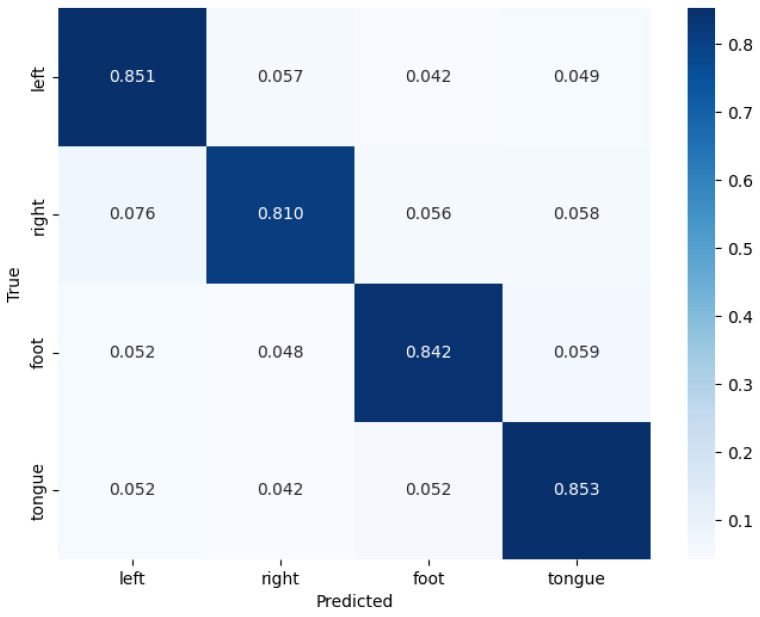
The confusion matrix of the model on the BCICIV-2a dataset for nine subjects.

On the BCICIV-2a dataset [44], the testing results of the ConSwinFomer model demonstrate the classification performance across different categories (left hand, right hand, both feet, tongue). The combined confusion matrix for nine subjects is shown in Figure 5. From the confusion matrix, it can be observed that for each category, the model exhibits a high classification accuracy rate. Especially for the first and fourth class (left hand motor imagery and tongue motor imagery), the correct classification rates reached 85.1% and 85.3%, respectively, and the misclassification rates to other classes were low. This indicates that the ConSwinFomer model performs well in capturing and distinguishing between different MI tasks. Similar to other studies, the classification performance of the right hand always appears to be the worst among these four categories, mainly due to being easily misclassified as the left hand.

As shown in Table 1, the accuracy of the model varies among different subjects. Subjects S03 and S07 achieved accuracies of 98.60% and 96.51%, respectively, showing extremely high classification performance. On the other hand, Subjects S02 and S05 exhibited relatively lower accuracies of 67.51% and 65.28%, which may be related to the specificity of the EEG signals of the subjects. The overall average accuracy was 83.99%, indicating that the ConSwinFomer model displays stable and efficient classification performance across different subjects.

### 4.5. Comparison

In our research, to validate the effectiveness of our proposed end-to-end ConSwinFormer network model, we conducted a series of comparative experiments, comparing our model with six current leading methods using the BCICIV-2a dataset. Below are the details of the comparative experiments.

The first method is FBCSP [9], a classic machine learning strategy that segments EEG signals into bands and applies CSP [8] within each sub-band for filtering and feature selection, followed by the classification procedure. The average classification accuracy of the FBCSP method is 16.57% lower than our model, indicating that while FBCSP achieves certain success in multi-band feature processing, it falls short in capturing complex EEG signal patterns.

Dong et al. [10] proposed PSCSP. In this method, artifacts in EEG signals are first removed by ICA, and then features are extracted using CSP. Six groups of spatial filters are constructed, and then the Gaussian kernel function and polynomial kernel function are combined into a hybrid kernel function. Finally, the hybrid kernel function is used to classify the extracted features. The classification accuracy of this method is 9.6% lower than ours. Using the ICA method requires a high level of expertise, and it displays similar problems to FBCSP, being unable to fully extract features in EEG signals.

Zhao et al. [26] proposed the Multi-branch 3D CNN model, adopting the 3D convolution of a multi-branch structure. Specifically designed to deal with the spatio-temporal characteristics of EEG data, it is the first model in the field of MI to start applying a 3D convolutional structure. Experimental results on BCICIV-2a show that the average classification accuracy of our ConSwinFormer model reaches 83.99%, which is significantly better than the 75.02% accuracy of the Multi-branch 3D CNN model. Our accuracy is 8.97% higher than theirs. Both our model and this model are based on 3D EEG data, and this result demonstrates the superior performance of our model in processing and classifying complex EEG signals.

Song et al. [19] proposed a Transformer-based spatio-temporal feature learning method using a Transformer representation in Table 1. After preprocessing, spatial filtering and employing the feature channel attention mechanism, the attention mechanism is used to train the model to perceive the global temporal dependence of EEG signals. Even though this method is similar to our model in structure, it is still 1.4% worse than our model regarding its classification effect.

The CNN-Transformer model proposed by Ma et al. [32] has some similarities to our model. They extract local features via a CNN and use a Transformer to obtain global features, which are then classified by softmax. Compared with this method, our model is innovative with regard to processing 3D data and capturing spatial features between channels more effectively, improving the classification effect by 0.08%.

Lastly, the CMO-CNN model proposed by Liu et al. [23] is a compact multi-branch one-dimensional convolutional network designed to extract features of MI tasks from raw EEG signals. Although the model performs convolution operations on multiple scales, its average accuracy is still 0.07% lower than our model.

These comparative experimental results demonstrate that our ConSwinFormer model surpasses other existing advanced methods in capturing and analyzing MI EEG signals. By combining the advantages of a CNN and a Swin Transformer, our model comprehends and decodes EEG signals more comprehensively, achieving higher classification performance in BCI tasks.

Table 1 shows the performance and average classification accuracy of each model on different subjects (S01 to S09). It also shows our model’s excellent performance on multiple subjects and on the overall average.

### 4.6. Ablation Experiments

To validate the effectiveness of each component of the ConSwinFormer model, we conducted ablation experiments. In our model, a CNN was utilized for local feature extraction, while a Swin Transformer was designed to capture global features. The ablation study involved removing the CNN and Swin Transformer parts, respectively, to verify their capabilities in extracting local and global features.

The results of the ablation experiments are displayed in Table 2. These results show the impact of omitting the CNN and Swin Transformer modules on accuracy across all test subjects. The ConSwinFormer model consistently outperforms models that only use a CNN or Swin Transformer. This demonstrates the efficacy of our approach of using a CNN to extract local features and a Swin Transformer to capture global information.

Table 2 provides detailed data on the performance comparison of the nine subjects, for which the ConSwinFormer model obtained the highest average accuracy of 83.99%. In comparison, the model using only the CNN achieved an average accuracy of 53.93%, and the model using only the Swin Transformer achieved an average accuracy of 77.85%. In all subjects’ ablation experiments, the ConSwinFormer model obtained the highest accuracy.

This ablation study clearly illustrates the complementary nature of the CNN and Swin Transformer modules in the ConSwinFormer model. The local feature extraction via the CNN and the global contextual understanding through the Swin Transformer together contribute to building a more robust and accurate MI-BCI model. The significantly higher average accuracy obtained by the ConSwinFormer model across all subjects further confirms the synergistic effect of combining these two powerful neural network mechanisms.

The spectral ablation experiments reveal three key insights for motor imagery decoding. As shown in Table 3, first, the α (9–12 Hz, 94.72%) and β (13–30 Hz, 95.26%) bands demonstrated superior performance. Second, while the combined α+β configuration achieved near-optimal accuracy (97.33%), its 1.27-percentage-point deficit compared to full-band processing (98.60%) confirms complementary information in other bands—particularly the θ rhythm’s temporal dynamics (4–8 Hz, 62.86%) that may encode preparatory neural states. Third, practical implementation requires computational trade-offs: resource-constrained systems could prioritize α+β bands (preserving 97.33% efficacy) with minimal performance sacrifices, while high-performance deployments should leverage full-spectrum analysis to maximize accuracy through cross-frequency interaction modeling. This dual-strategy framework balances biological plausibility with engineering pragmatism, as δ/θ bands (0.5–8 Hz) become critical only when pursuing marginal accuracy gains, which justifies their computational overhead.

### 4.7. Parameter Sensitivity

In our research, we have found that the performance of the model is significantly influenced by some key parameters. To explore how these parameters affect the model’s performance in detail, we conduct a series of in-depth experiments. This section will describe and discuss the impact of the depth of the Swin Transformer in detail, including the number of attention heads, the size of data divided into patches, and the size of the CNN convolution kernel on the temporal dimension.

**Figure 6:**
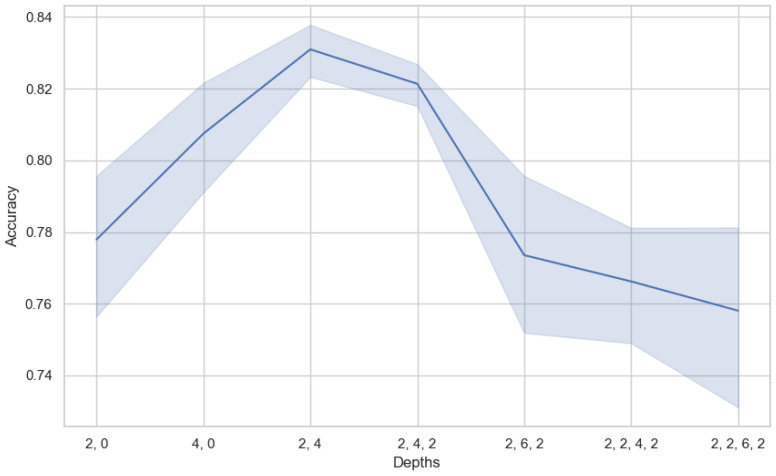
The impact of model depth on performance.

Similar to the CNN, the Swin Transformer model is designed to extract data features at various scales by stacking multiple layers. Thus, the depth of the model, or the number of layers, determines the number of levels at which the model can extract features, directly affecting the model’s learning ability and feature extraction effectiveness. Increasing the model’s depth can enhance the model’s ability to abstract and represent data features, especially when dealing with complex time-series data. However, too deep a model may lead to overfitting and increased training difficulty. Therefore, by comparing the performance of models under different depth parameters, we aim to find the optimal parameters, which is crucial for end-to-end training performance. We selected seven model depth parameters, as shown in Figure 6, where the commas separate the number of layers, and the numbers represent the number of modules in that layer. Since each layer contains at least two modules (3DW-MSA, 3DSW-MSA) in the Swin Transformer, each layer model has an even number of MSA blocks.

Through the analysis of the visualization of depth parameters’ influence on the model’s accuracy, we observe that the model is sensitive to the depth of different hierarchical structures. It can be clearly seen from the figure that the accuracy of the model is lower when the depth parameter of the model is at a shallow level (such as (2, 0)). As the depth increases to (2, 4), the model acquires a second layer. The first layer contains a Swin Transformer block, and the second layer contains two Swin Transformer blocks. At this depth parameter, the model accuracy reaches its highest point. However, increasing the depth does not improve the accuracy. Instead, it results in a decrease in accuracy and an increase in variance, suggesting that the model may have suffered from overfitting. It can be seen that the proper model depth is crucial for model performance. In our experiments, the parameter with a depth of (2, 4) provides the best performance. This parameter achieves effective feature extraction and learning by reasonably arranging the number of Swin Transformer modules at different levels. There is no need to concern ourselves with the over-fitting or high complexity of the model, since the number of parameters is small and the inference results can be obtained in a short time.

**Figure 7:**
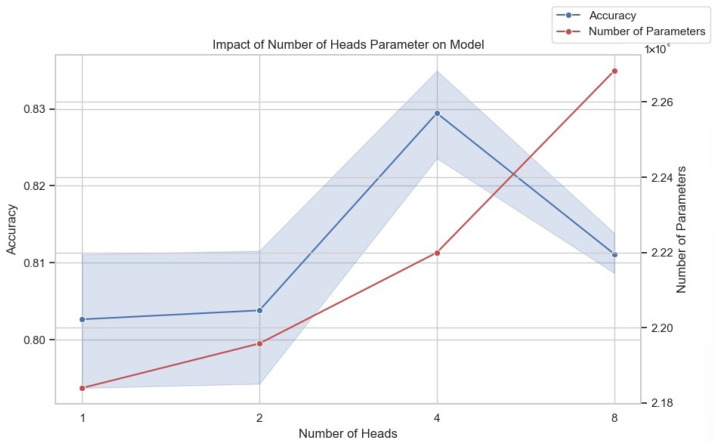
The impact of the number of heads in MSA on model performance.

In deep learning models, the multi-head attention mechanism enhances the model’s ability to process input data by allowing it to attend to information from multiple distinct representational subspaces simultaneously. This parallel processing enables the model to capture diverse aspects and features of the data more comprehensively, often leading to improved performance on complex tasks. However, incorporating multiple attention heads significantly increases the model’s parameter count, necessitating a careful trade-off between model performance and computational resources during training and deployment. Figure 7 illustrates the impact of varying the number of attention heads on both model accuracy and the total number of parameters. Our experiments evaluated model configurations with one, two, four, and eight attention heads. As shown, starting with one head, the model achieved baseline accuracy. Increasing the number of heads to two resulted in a modest improvement in accuracy. A more substantial increase in accuracy was observed when the number of heads was further increased to four, reaching the peak performance among the tested configurations. However, increasing the number of heads to eight led to a decrease in accuracy compared to the four-head configuration. Simultaneously, the figure clearly demonstrates that the number of model parameters increases significantly with each increment in the number of attention heads. Given the observed performance trend, where accuracy peaks at four heads and declines thereafter, combined with the substantial increase in computational complexity indicated by the ballooning parameter count, we determined that using four attention heads offers the most favorable balance between model performance and efficiency for our specific requirements and available computational resources. Further increasing the number of heads beyond four proved detrimental to accuracy while incurring a significant additional computational burden.

**Figure 8:**
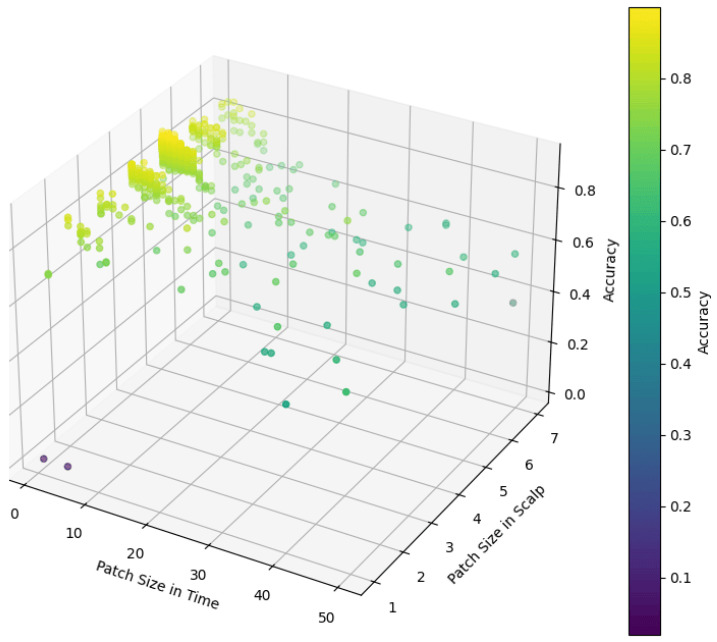
The impact of patch size on model performance.

In our model, the role of the patch size parameter is to divide the input data into multiple small patches, which are used to separate the computational units, namely tokens, in the model for subsequent feature extraction and analysis. To explore the effect of patch size on model performance, we conduct an exhaustive series of experiments. As shown in Figure 8, we tested different patch size settings in temporal and scalp spaces. Experimental results show that in the temporal dimension, when the sampling point is set to 2, the model can achieve high accuracy. This indicates that fewer temporal sampling points are sufficient to capture effective temporal features and provide effective information for the model. It is also noticed that the accuracy of the model drops sharply when the time sampling points exceed 5. This may be because too many time sampling points lead to the loss of local information of the time series, or increase the complexity of the model, which affects the generalization ability of the model. In the scalp space, we find that setting the patch size to (5×5) enables us to achieve the best results. This finding indicates that on the scalp space, the model optimizes performance by extracting global information rather than local details, which provides important features for subsequent classification tasks. This extraction of global information may be related to the spatially distributed properties of brain activity patterns, where synchronous activity in larger regions is more critical for distinguishing different MI tasks than local activity in smaller regions.

**Figure 9:**
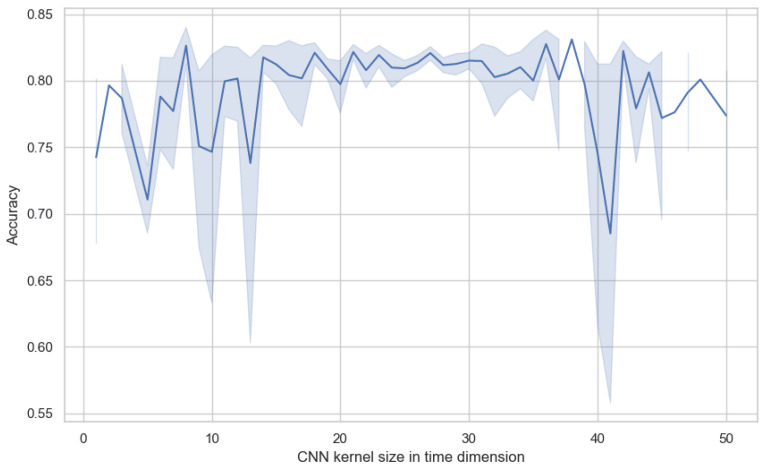
The impact of temporal kernel size in the CNN module on model performance.

In our model, the primary task of the convolutional neural network (CNN) is to extract preliminary local features in the spatial and temporal dimensions. We not only referred to the parameter recommendations provided in [40] but also extensively experimented with the size of the CNN convolution kernel in the temporal dimension. As shown in Figure 9, the experimental results show that the parameter with the size of a convolution kernel has a direct impact on model performance. If the convolution kernel size is set to a value that is too large or too small, it adversely affects the model’s effectiveness, as indicated by decreased accuracy and increased variance. Too small a convolution kernel may fail to capture sufficient temporal information. On the other hand, too large a kernel might lead to excessive merging of temporal information, thereby losing important temporal details. Through extensive experimental comparisons, we found that setting the convolution kernel size to between 25 and 30 on the temporal dimension allows the model to achieve optimal performance. Within this range, the convolution kernel is large enough to capture key temporal features in EEG signals while avoiding unnecessary noise and redundant information interference. Additionally, this convolution kernel size also maintains the model’s variance at a lower level, meaning that the model’s performance is more stable and does not fluctuate dramatically due to minor changes in input data.

## 5. Visualization

**Figure 10:**
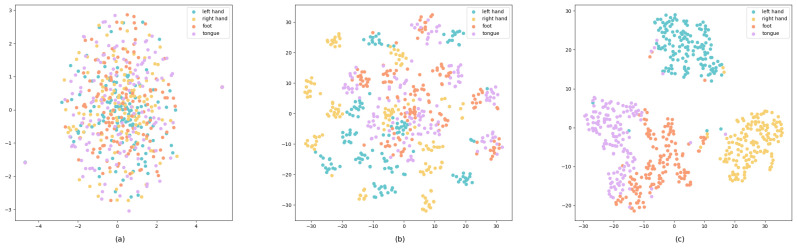
t-SNE visualization (**a**) after preprocessing, (**b**) after the CNN module, and (**c**) after the Swin Transformer module.

In our research, the t-SNE (t-distributed Stochastic Neighbor Embedding) [48] technique is used for a dimensionality reduction and visualization [48]. It is used here to intuitively show the feature extraction and classification effects of the model in different processing stages, and to enhance the interpretability of the model. Moreover, it is a highly popular machine learning algorithm capable of effectively mapping high-dimensional data to a low-dimensional space for visualization. We applied the t-SNE to data from three key stages in the model: after preprocessing, after processing via the CNN module, and before the classification module. As shown in Figure 10, Figure 10a shows the distribution of samples after only preprocessing, with the samples being mixed with each other and there being no obvious clustering trend, indicating that the features of different categories have not been effectively distinguished at this stage. Figure 10b shows the samples processed by the CNN module, with the samples showing a certain degree of an aggregation effect. It also shows that the CNN module has extracted some features that are helpful for distinguishing different categories in the spatial-temporal dimension, but there are still some cases that are unclearly classified. Figure 10c shows the distribution of samples before the final classification. The distribution of samples after the 3D Swin Transformer module is used shows a significant clustering effect. The distance between samples of different classes becomes more distinct. This shows that the 3D Swin Transformer module can effectively extract representative features and further strengthen the discriminative ability of the model.

It is particularly noteworthy that in these visualizations, the distribution of data points for the left and right hands is far apart, indicating the model’s strong ability to distinguish between these two categories. However, there is some overlap between the data points representing the right hand and both feet, showing that some samples are classified as fuzzy. The data points for the tongue and both feet, despite not being too far apart, exhibit a clear boundary line in the visualization, indicating that these two categories can also be effectively distinguished.

By utilizing gradients calculated during specific layers of the model, we employ the GradCAM algorithm [49] to determine the contribution of each token to the classification result. These contributions are then mapped back to the spatial locations of the original EEG data, forming a 3D heat distribution. We divide the feature map into five segments, we overlay each time segment on the time dimension, and we plot it as a topographic map. This was completed to more clearly visualize which brain regions the model paid more attention to at different times. Additionally, we also projected the 3D heat distribution onto the temporal dimension to show which parts of time the model paid more attention to.

**Figure 11:**
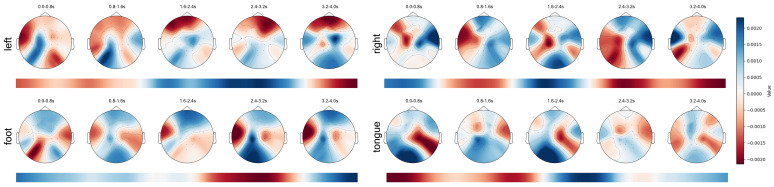
Spatio-temporal feature maps for four MI paradigms of Subject 3.

Figure 11 presents the spatio-temporal feature maps of Subject 3 in four different categories of MI tasks. We can observe that the data at the start of the experiment show widespread suppression, possibly indicating a certain latency in the initiation of MI. Furthermore, we find a correlation between the speed of the activation response and the distance to the brain center: areas closer to the brain center respond more swiftly, such as the motor cortex’s reaction to controlling the tongue. In the spatial dimension, the primary activation areas related to MI tasks are concentrated in the motor cortex, consistent with the motor paradigm [50]. During the imagination of the left and right hand movements, we observe a clear contralateral activation and ipsilateral suppression, aligning with the phenomena of event-related desynchronization (ERD) and event-related synchronization (ERS) [51,52]. Temporally, the execution process of an action does not always maintain the same activation areas but undergoes continuous changes. Some activation areas’ location shifts or they show gradually increasing or decreasing phenomena. We believe this indicates that an MI paradigm may involve multiple brain areas working together. Specifically, multiple brain areas participate in coordinating an MI paradigm at different times, and the activation location of a brain area changes over time.

## 6. Discussion

In this research, we introduced a novel model and tested it on the BCICIV-2a dataset. The results show that the model performs well in the classification of MI tasks. By analyzing the EEG data from different subjects, we observed significant differences in accuracy among individuals. This variation is likely related to the uniqueness of each subject’s EEG signals, especially for Subjects S03 and S07, achieving accuracies of 98.60% and 96.51%, respectively. This suggests that these subjects’ EEG signal patterns during MI tasks have higher distinguishability, enabling more effective classification by the model.

A detailed analysis of the confusion matrix revealed that classification errors mostly occurred between right hand MI and tongue MI. This confusion may stem from the high similarity in EEG signal patterns between these two tasks, posing a challenge to the model’s discriminative ability. Moreover, our model achieved an average classification accuracy of 83.98% across all subjects, surpassing many other current methods and proving its effectiveness in capturing EEG signal features.

In terms of model visualization analysis, the spatio-temporal feature maps suggest that multiple brain regions might be involved in coordinating and scheduling tasks during an MI task at different times. Based on this observation, we propose a new research direction: incorporating connectivity features between brain regions on top of the existing spatio-temporal frequency model. We preliminarily consider the extracted feature maps as representative points of brain regions, while the connectivity strength between regions is regarded as edge weights, forming a graph containing both spatio-temporal frequency and brain region connectivity features. We plan to further explore this concept in future research.

Considering the needs of practical BCI applications, such as assisted communication and neurological rehabilitation, our model has the potential to provide users with more precise control capabilities due to its excellent accuracy and stability. Due to these qualities, there is a prospect of integrating the model into real-time BCI systems, providing effective communication and control of peripherals for individuals with limited mobility. In order to further improve the practicality and applicability of the model, our future research work will implement the model in a wider group of subjects, as well as verify and test the model under complex noise conditions in the real world.

Although this research achieved some good results, we also clearly recognize the model’s limitations. In our future work, we will explore more data augmentation methods to mitigate issues associated with small datasets and further reduce the risk of overfitting. Considering the individual differences in EEG signals among subjects, we will also investigate personalized adjustment strategies to enhance the model’s generalizability across subjects. Furthermore, we will further study the model’s potential for multitask learning and transfer learning to improve its adaptability and accuracy across different EEG signal tasks.

## 7. Conclusions

In this research, we propose an innovative deep learning model that combines a CNN and a Swin Transformer. The aim is to improve the classification accuracy of EEG signals for MI tasks. Firstly, the model utilizes the powerful ability of the CNN in capturing the local spatio-temporal features of EEG data, and combines the advantages of the Swin Transformer in global information processing to achieve efficient classification of EEG signals. Through a series of experiments on BCICIV-2a, the average accuracy reaches 83.99%. Secondly, in individual subjects, the accuracy of the model is even as high as 98.60%, which strongly demonstrates the stability and accuracy of our model. Finally, our model shows a significant performance improvement compared to existing state-of-the-art models, both traditional machine learning methods such as FBCSP [9] and recent Transformer-based deep learning methods [18,31]. This finding further confirms the capability of deep learning, especially the strategy of the CNN combined with the Swin Transformer, in dealing with complex data, such as EEG data.

In addition, data augmentation techniques are used during model training. Specifically, our adopted the segmentation and reconstruction (S&R) technique and random noise addition. This significantly improved the model’s ability to generalize small datasets and reduced the risk of overfitting. The prediction ability of the model on new data was enhanced, and the robustness of the model was effectively improved.

In summary, this research not only theoretically proposes an effective MI classification model but also experimentally verifies its effectiveness in practical BCI applications. In the future, we will conduct further tests and test the model on some larger and diverse datasets. We will also explore its application to different BCI tasks and extend our research to a broader field of neuroscience and clinical practice.

## Figures and Tables

**Table 1:** The model we proposed is compared with the most advanced model on the BCICIV-2a dataset. The best score is displayed in bold.

	S01	S02	S03	S04	S05	S06	S07	S08	S09	Average
FBCSP [9]	76.00	56.50	81.25	61.00	55.00	42.25	82.75	81.25	70.75	67.42 ± 13.54
PSCSP [10]	80.00	65.36	87.14	67.50	55.54	50.18	91.79	84.11	87.86	74.39 ± 14.31
Multi-branch 3D CNN [26]	77.38	60.14	82.93	72.29	75.84	68.99	76.04	76.86	84.67	75.02 ± 6.92
Transformer [19]	91.67	71.67	95.00	**78.33**	61.67	66.67	96.67	93.33	88.33	82.59 ± 12.52
CNN-Transformer [32]	92.02	**78.12**	95.13	78.30	64.23	67.88	**97.03**	93.23	89.23	83.91 ± 11.49
CMO-CNN [23]	86.93	67.47	92.69	77.21	**82.78**	**73.73**	92.52	90.43	**91.47**	83.92 ± 8.69
Our	**93.76**	67.51	**98.60**	76.17	65.28	72.64	96.51	**94.70**	90.72	**83.99 ± 12.64**

**Table 2:** The results of the CNN and Swin Transformer modules were extracted from ConswinFormer for ablation experiments.

	S01	S02	S03	S04	S05	S06	S07	S08	S09	Avg
CNN	54.03	51.39	52.77	56.80	52.77	54.16	59.87	53.35	50.26	53.93
Swin Transformer	90.01	51.38	97.34	72.98	51.95	65.00	92.93	93.33	88.76	77.85
ConSwinFormer	**93.76**	**67.51**	**98.60**	**76.17**	**65.28**	**72.64**	**96.51**	**94.70**	**90.72**	**83.99**

**Table 3:** Accuracy comparison (%) of spectral band ablation experiments for Subject 03.

δ (0.5–4 Hz)	θ (4–8 Hz)	α (9–12 Hz)	β (13–30 Hz)	γ (31–50 Hz)	α+β	Full-Band
53.61	62.86	94.72	95.26	47.28	97.33	**98.60**

## Data Availability

Data is contained within the article.
